# Screening for depression in women during pregnancy or the first year postpartum and in the general adult population: a protocol for two systematic reviews to update a guideline of the Canadian Task Force on Preventive Health Care

**DOI:** 10.1186/s13643-018-0930-3

**Published:** 2019-01-19

**Authors:** Candyce Hamel, Eddy Lang, Kate Morissette, Andrew Beck, Adrienne Stevens, Becky Skidmore, Heather Colquhoun, John LeBlanc, Ainsley Moore, John J. Riva, Brett D. Thombs, Ian Colman, Sophie Grigoriadis, Stuart Gordon Nicholls, Beth K. Potter, Kerri Ritchie, Julie Robert, Priya Vasa, Bianca Lauria-Horner, Scott Patten, Simone N. Vigod, Brian Hutton, Beverley J. Shea, Shamila Shanmugasegaram, Julian Little, David Moher

**Affiliations:** 1 Knowledge Synthesis Group, Clinical Epidemiology Program, Ottawa Hospital Research Institute, Ottawa, Ontario Canada; 2 University of Calgary Cumming School of Medicine, Calgary, Alberta Canada; 3 Alberta Health Services, Calgary, Alberta Canada; 4 Public Health Agency of Canada, Ottawa, Ontario Canada; 5 Department of Occupational Science and Occupational Therapy, University of Toronto, Toronto, Canada; 6 Department of Pediatrics, Dalhousie University, Halifax, Nova Scotia Canada; 7 Department of Health Research Methods, Evidence and Impact, McMaster University, Hamilton, Canada; 8 Department of Family Medicine, McMaster University, David Braley Health Sciences Centre, Hamilton, Canada; 9 Lady Davis Institute of the Jewish General Hospital, Montreal, Quebec Canada; 10 Department of Psychiatry, McGill University, Montreal, Quebec Canada; 11 School of Epidemiology and Public Health, Faculty of Medicine, University of Ottawa, Ottawa, Ontario Canada; 12 Department of Psychiatry, University of Toronto, Toronto, Canada; 13 Sunnybrook Health Sciences Centre, Toronto, Canada; 14 Women’s College Research Institute, Women’s College Hospital, Toronto, Ontario Canada; 15 Ottawa Hospital Research Institute, Ottawa, Ontario Canada; 16 School of Psychology, University of Ottawa, Ottawa, Ontario Canada; 17 Department of Psychology, The Ottawa Hospital, Ottawa, Ontario Canada; 18 Patient representative, Ottawa, Ontario Canada; 19 Department of Family and Community Medicine, St. Michael’s Hospital, University of Toronto, Toronto, Ontario Canada; 20 Department of Psychiatry, Dalhousie University, Halifax, Nova Scotia Canada; 21 Department of Community Health Services and Department of Psychiatry, University of Calgary, Calgary, Alberta Canada; 22 Centre for Journalology, Clinical Epidemiology Program, Ottawa Hospital Research Institute, Ottawa, Ontario Canada

**Keywords:** Depression, Screening, Systematic review, Adults, Pregnancy, Postpartum

## Abstract

**Background:**

In 2018, the World Health Organization reported that depression is the most common cause of disability worldwide, with over 300 million people currently living with depression. Depression affects an individual’s physical health and well-being, impacts psychosocial functioning, and has specific negative short- and long-term effects on maternal health, child health, developmental trajectories, and family health. The aim of these reviews is to identify evidence on the benefits and harms of screening for depression in the general adult population and in pregnant and postpartum women.

**Methods:**

Search strategies were developed and tested through an iterative process by an experienced medical information specialist in consultation with the review team. We will search MEDLINE, Embase, PsycINFO, CINAHL, and the Cochrane Library, and a randomized controlled trial filter will be used. The general adult review will be an update of a systematic review previously used by the Canadian Task Force on Preventive Health Care for their 2013 guideline recommendation. The search strategy will be updated and will start from the last search date of the previous review (May 2012). The pregnant and postpartum review will be a de novo review with no date restriction. For both reviews, we will search for unpublished documents following the CADTH Grey Matters checklist and relevant websites. Titles and abstracts will be screened using the liberal accelerated method. Two reviewers will independently screen full-text articles for relevance using pre-specified eligibility criteria and assess the risk of bias of included studies using the Cochrane Risk of Bias tool. Outcomes of interest for the general adult population review include symptoms of depression or diagnosis of major depressive disorder, health-related quality of life, day-to-day functionality, lost time at work/school, impact on lifestyle behaviour, suicidality, false-positive result, labelling/stigma, overdiagnosis or overtreatment, and harms of treatment. Outcomes of interest for the pregnant and postpartum review include mental health outcomes (e.g. diagnosis of major depressive disorder), parenting outcomes (e.g. mother-child interactions), and infant outcomes (e.g. infant health and development).

**Discussion:**

These two systematic reviews will offer informative evaluations of depression screening. The findings will be used by the Task Force to help develop guideline recommendations on depression screening in the general adult population and in pregnant and postpartum women in Canada.

**Systematic review registration:**

PROSPERO (CRD42018099690)

**Electronic supplementary material:**

The online version of this article (10.1186/s13643-018-0930-3) contains supplementary material, which is available to authorized users.

## Introduction

Depression is a mood disorder characterized by states of sadness and feelings of worthlessness or emptiness and accompanied by physical symptoms such as decreased activity, poor appetite, and poor sleep serious enough to impair functioning in social, occupational, educational, or other situations [1]. The current definition of a major depressive episode (MDE) is based on one of two classifications [2]: DSM-5 [3] and ICD-10 [4]. The DSM-5 includes additional criteria to define major depressive disorder (MDD) (see Additional file 1). The DSM-5 allows for a specifier for depressive episodes that have their onset in pregnancy or within 4 weeks postpartum, collectively termed major depressive episodes, with peripartum onset. Of note, a woman can still meet criteria for depression in pregnancy or postpartum even if the onset did not occur within the “peripartum onset” time frame. In clinical practice and research, depression occurring up to 1 year postpartum is generally considered “postpartum depression” [5].

### General adult population

#### Prevalence

Depression is the most common cause of disability worldwide, with over 300 million people now living with depression, an increase of more than 18% between 2005 and 2015 [6]. Estimates of prevalence for depression vary by characteristics such as age and sex. For example, women are more likely to suffer from major depressive disorders than men [7, 8]. Many studies report depression rates based on results from self-reported screening questionnaires, rather than validated diagnostic interviews, but this is known to exaggerate rates substantially and to blur distinctions between low- and high-prevalence groups [9]. The 2012 Canadian Community Health Survey-Mental Health used the diagnostic interview technique among 25,113 individuals and reported annual prevalence for major depressive disorder (MDD) of 3.9% (95% CI 3.5–4.2%) and lifetime prevalence of 9.9% (95% CI 9.3–10.5%) [10]. It also reported an annual and lifetime prevalence of MDE among Canadians at 4.7% and 11.3%, respectively [10]. Another 2012 Canadian national health survey reported the highest rate of a MDE was among 15–24-year-olds, with 7% having had depression in the past year, compared to 5% in people aged 25–64 years, and 2% in those 65 years and older [11].

#### Risk factors

There are several risk factors that have been associated with depression in adults. Socio-demographic risk factors include sex, age, marital status, low socioeconomic status, and low education level [8, 12–14]. In Canada, the largest difference between sexes is in the 15–24 age range, with the difference diminishing and nearly disappearing at more advanced ages [15]. Additionally, married and never-married individuals experience less depression than those who are separated, divorced, and widowed [8]. Other factors such as trauma early in life (e.g. neglect or sexual abuse), chronic disease (e.g. cancer, cardiovascular disease), previous history of depression, and a family history of depression have also been linked to depression [8, 16, 17].

#### Consequence of depression

Depression affects a person’s physical health and well-being and impacts psychosocial functioning (e.g. personal relationships, employment). A review by Evans et al. [18] conclude that there may be a bidirectional link between depression and disease, as depression might be an etiologic factor for new disease (e.g. stroke) and also might affect the course of existing chronic diseases such as diabetes mellitus. Depression can affect work performance through absenteeism and presenteeism (decreased work productivity while at work), which is a large cost to employers in terms of productivity [2]. In addition, many depressed individuals are unable to enter the workforce. On a population level, it also has a large societal impact through increased health service utilization, decreased work productivity, increased burden on family members, and increased resource costs related to disability [19]. In the 2003 Canadian Community Health Survey, the total economic burden of mental illness (including health service utilization, long- and short-term work loss, and health-related quality of life) was said to be $51 billion dollars [19]. More recently, direct healthcare costs associated with MDD were determined using a population-based cohort study in Ontario, Canada. The age- and sex-adjusted annual per-capita cost among those with MDD was higher than the comparison group (those without MDD or psychological distress) [$3914 (95%CI $2943–4888) vs $3206 (95%CI $2820–3591)], and the population-wide excess cost for those with MDD was $256 million (prices converted to CDN $ from reported USD) [20].

Although effective interventions to reduce the effects of depression exist, individuals need to be identified to benefit from these interventions. The Mental Health Commission of Canada reports that almost half of those who feel they have suffered from depression or anxiety have not seen a doctor about this problem [21]. In addition, among those who have been diagnosed accurately, many do not receive minimally adequate treatment [21, 22].

#### Current recommendations

In 2013, the Canadian Task Force on Preventive Health Care (CTFPHC) recommended to not routinely screen for depression (this was based on very-low-quality evidence; see Additional file 2). There is disagreement in recommendations between Canada, the USA, and the UK. Neither the CTFPHC nor the United Kingdom National Screening Committee (UKNSC) recommended screening, whereas the US Preventive Services Task Force (USPFTF) recommended screening based on prioritization of linked evidence of effective follow-up and treatment of screen-identified individuals. Additional file 2 provides additional on how the USPSTF recommendation differs from Canada and the UK, followed by some speculation as to why [23].

### Pregnant and postpartum population

#### Prevalence

Although estimates of the prevalence of major depression should be based on validated diagnostic interviews, many studies report depression rates based on results from self-reported symptom questionnaires and other non-valid methods [9]. An Agency for Healthcare Research and Quality SR reported that the period prevalence of major depression during pregnancy (conception to birth) was 12.7% (95%CI 7.1–20.4%) [24]. However, this is based on one primary study. The period prevalence from birth to 3 months postpartum was 7.1% (4.1–11.7%) [24]. A recent US study in which women were interviewed, and diagnosis made using the DSM-IV criteria, found the 12-month period prevalence of MDD to be 8.4% among women who were currently pregnant or had been pregnant in the past 12 months, 9.3% among postpartum women, and 8.1% among non-pregnant women [25]. It should be noted that the prevalence for postpartum women could include time in which they were pregnant, as it covers the previous 12 months.

#### Risk factors

There are many risk factors for depression during pregnancy, including younger age, a history of depression, exposure to domestic violence, increased life stressors, a lack of social support, unintended pregnancy, lower income, lower education, smoking, single status, and poor relationship quality [26, 27]. Prior depression is the greatest risk factor for postpartum depression. Nevertheless, for women who experience postpartum depression, it is a first episode among 40% [28]. Other postpartum risk factors include untreated depression or anxiety during pregnancy, experiencing a stressful life event during pregnancy, having a traumatic birth experience, preterm birth or infant admission to neonatal intensive care, low levels of social or partner support, experiencing domestic violence, low socioeconomic status, obstetric complications, low birth weight, and breastfeeding problems [27].

#### Consequence of pregnancy and/or postpartum depression

While the prevalence of depression in women during pregnancy and the first year postpartum may be similar to that for other women [24], depression has specific negative short- and long-term effects on maternal health, child health and development, and on the overall health of families [29]. Depression during pregnancy is associated with unhealthy behaviours including poor self-care, poor nutrition, increased use of tobacco and alcohol, lower prenatal care seeking, and poorer maternal-fetal bonding [30, 31]. Postpartum depression may lead to difficulties with infant care, a decrease in breastfeeding initiation, and poor-quality mother-child interactions including mutual touching, smiling, and vocalizations, and compromised mother-child bonding [30, 32, 33]. Negative outcomes for infants in mothers with prenatal and postpartum depression may also include preterm delivery, lower birth weight, cognitive, emotional, social, neural functioning or developmental delay [34–37].

Almost half of Canadians with depression have not seen a primary care provider about their depression [38]; for depression in pregnancy and postpartum, the number may be even higher [35]. Screening for depression, if effective, would allow for treatment among women who would not otherwise be identified and possibly lessen the negative impacts to the mother, fetus/infant, and family. Several treatment options exist, including psychosocial strategies (e.g. peer support, non-directive counselling and self-care such as exercise), psychological therapies, and antidepressant medications [36, 37]. The last poses the additional challenge of considering the safety of exposure to psychotropic medications to the baby in utero and through breast milk [39].

#### Current practice and recommendations

Across Canada, there is a lack of consensus on how and when prenatal and postpartum depression screening should occur with different provinces and territories having different approaches. Additional file 2 provides examples on how the provinces of Ontario, British Columbia, Alberta, Nova Scotia, and the territory of Nunavut screen women during pregnancy and postpartum. There is discordance in recommendations between Canada, the USA, and the UK. Neither the CTFPHC or UKNSC recommended screening in contrast to the USPFTF recommendation for screening on results that combined screening with treatment. Additional file 2 provides additional details on why the USPSTF recommendation may differ from Canada and the UK [23].

### Definition of a controlled trial of screening intervention

The intent of a screening programme for depression would be to identify symptomatic disease that would not otherwise be identified or reported (i.e., by spontaneous patient self-report or clinical inquiry). Current approaches for depression screening are based on the use of questionnaires (e.g. Edinburgh Postnatal Depression Scale (EPDS), Patient Health Questionnaire (PHQ-9), Beck Depression Inventory) to identify people who may have undetected depression. If effective, screening for depression could reduce the health burden in those who otherwise would not be identified [23].

The following three eligibility criteria have been used when considering depression screening trials [40]: (i) the patient population must be clearly defined and participants randomized prior to administering the screening test; (ii) patients who are known to have a current episode of depression or are already being treated for depression close to the time of eligibility assessment are excluded, as screening is intended to identify undetected cases and those who are known to have depression would not be screened in actual clinical practice; and (iii) similar depression management and treatment resources must be provided to patients in the screening arm of the trial and patients in the non-screening arm of the trial who are identified as depressed via other methods (e.g. unaided clinician diagnosis, patient report).

### Objective

**Fig. 1:**
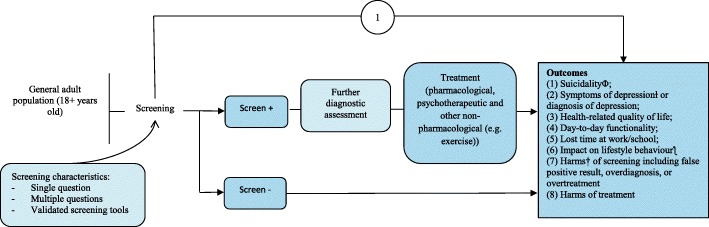
Analytic framework for depression screening in the general adult population

**Fig. 2:**
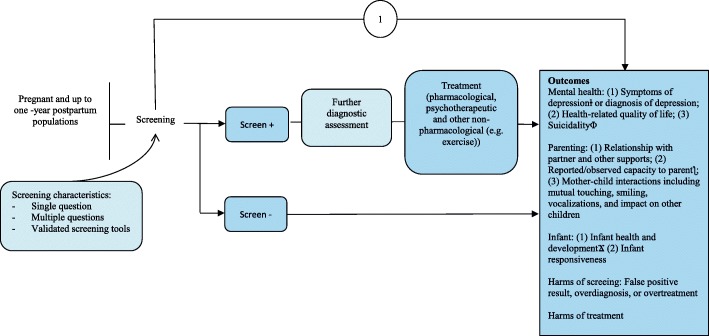
Analytic framework for depression screening in pregnant and postpartum women

The CTFPHC is undertaking a systematic evaluation of the evidence to inform its guideline recommendations for depression screening during pregnancy and up to 1 year postpartum in primary health care settings in Canada and to provide an updated recommendation for the general adult population. This protocol outlines the methodological process for performing these two systematic reviews (SR) of the evidence on the benefits and harms of screening for depression. This protocol updates the 2013 McMaster Evidence Review and Synthesis Centre (ERSC) SR previously used by the CTFPHC [41] for their guideline recommendation on depression screening in adults [42], where the pregnant and postpartum population was considered as a subgroup of the general adult population. The scope of the forthcoming guideline has been revised to more formally consider women during pregnancy and postpartum. The analytic framework depicts the structure used to address the key questions for evaluating the benefits and harms of depression screening (see Figs. 1 and 2). We will use the following key questions to guide the SRs.

#### General adult population

##### Key question 1

What are the benefits and harms of screening versus no screening for depression in the general adult population in primary care or other non-mental health clinic settings?

##### Key question 1a

What are the benefits and harms of screening versus no screening for depression in the general adult population in primary care or other non-mental health clinic settings for patients targeted because they have characteristics that may suggest an elevated risk of depression? (characteristics as defined in primary studies, not including exclusion criteria).

#### Pregnant and postpartum population

##### Key question 1

What are the benefits and harms of screening versus no screening for depression during pregnancy and up to 1 year postpartum in primary care or other non-mental health clinic settings?

##### Key question 1a

What are the benefits and harms of screening versus no screening for depression during pregnancy and up to 1 year postpartum in primary care or other non-mental health clinic settings for patients targeted because they have characteristics that may suggest an elevated risk of depression? (characteristics as defined in primary studies, not including exclusion criteria (e.g. previous depression in pregnancy or postpartum)).

This systematic review is being conducted to inform a guideline on screening for depression. We will conduct a separate systematic review on additional key questions about patient values and preferences should the working group decide it is needed to inform the guideline. For each population, after reviewing the evidence from KQ1 and KQ1a, if the working group believes that SR information on patient values and preferences would potentially change recommendations beyond what is learned about values and preferences identified from focus groups conducted by the Knowledge Translation Team of St. Michaels Hospital in Toronto, Ontario [43], supporting the development of recommendations for this guideline, then we will move forward with this additional review(s). The potential key questions are:

##### Key question 2

How do patients value outcomes that may occur from screening for depression in the general adult population and how do these values influence decisions about being screened?

##### Key question 2a

How do patients with characteristics that may suggest an elevated risk of depression value outcomes that may occur from screening for depression in the general adult population and how do these values influence decisions about being screened? (characteristics as defined in primary studies, not including exclusion criteria).

The same key questions on patient values and preferences may be addressed in the pregnant and postpartum population. The decision to proceed or not proceed in one population does not determine whether patient values and preferences will be undertaken for the other population. If we do pursue a SR on KQ2 and KQ2a, a separate protocol will be developed at that time. This would include topic refinement and all relevant Population, Intervention, Comparator, Outcome (PICO) criteria and methods.

## Methods

These SRs will be developed, conducted, and prepared according to the CTFPHC Procedure Manual [44]. The manual is a living document and if any changes to the current methods occur, they will be reported in the full review. A Depression Working Group of CTFPHC members was formed for the development of the topic, refinement of the key questions and scope, and rating of outcomes considered most important for creating a recommendation; this working group also sought input from external clinical and content experts. For more information on the selection of working group members and clinical experts, please refer to the CTFPHC Procedure Manual (https://canadiantaskforce.ca/methods/). We have invited patients to partner with the team to gain from their perspectives and learn from their knowledge regarding the prioritization of the outcomes. The general adult review is an update of a SR [41] previously used by the CTFPHC for their 2013 guideline recommendation on depression screening in adults [42]. Phrasing of the key questions and the eligibility criteria (i.e., PICOS) were also reviewed, re-evaluated, and modified where required (e.g. outcomes). The Depression Working Group has developed the list of outcomes that were rated according to the GRADE methodology [45]. Through consensus, outcomes rated as critical for decision-making (rated 7 to 9 out of 9) and important (rated 4 to 6 out of 9) are included. In addition, these outcomes were rated by patients as well as other outcomes deemed important to patients identified through the patient engagement activities conducted by the Knowledge Translation Program at St. Michael’s Hospital in Toronto, Ontario. Patients rated all patient-important outcomes as critical (7 to 9 out of 9) or important (4 to 6 out of 9) for decision-making. The list of outcomes was finalized after the input from patients.

This SR protocol was prepared in accordance with the PRISMA-P guidelines [46] (see Additional file 3) and registered with the International Prospective Registry of Systematic Reviews (PROSPERO) database (CRD42018099690). The reviews will be reported according to the PRISMA statement [47] and will include a PRISMA flow diagram. We will also use the conduct reported in a Measurement Tool to Assess the Methodological Quality of Systematic Reviews (AMSTAR 2) [48] tool for additional quality control. Any amendments made to this protocol when conducting the reviews will be outlined in the related review’s manuscript.

### Eligibility criteria

Studies for each review will be selected according to the inclusion and exclusion criteria in Tables 1 and 2.

**Table 1:** Criteria for inclusion and exclusion of studies in the general adult review

	Inclusion criteria	Exclusion criteria
Population	Key question 1: patients who are 18 years and older Key question 1a: patients who are 18 years and older selected for screening because they have characteristics that may suggest an elevated risk of depression* *Characteristics as defined in primary studies (e.g. trauma early in life, a family history of depression)	- If > 20% of the study population have a recent history of depression, have a current diagnosis, or are receiving treatment for depression or other mental disorders (unless results are provided separately from the population of interest) - Seeking services due to symptoms of mental disorders - Receiving assessment or care in psychiatric or mental health settings
Intervention	Interventions that use a single question, small sets of questions, or a screening questionnaire (validated or non-validated) with a pre-defined cut-off score to identify patients who may have depression, but who have not reported their symptoms to healthcare providers or who have otherwise not been identified as possibly depressed by healthcare providers	Interventions that, in addition to screening, include depression care referral or treatment options that are not available to patients identified as depressed in the non-screening trial arm are excluded.
Comparator	No depression screening Patients in comparator trial arms may be administered depression symptom questionnaires for the purpose of baseline or outcome assessments as long as scores are not provided to the patients or healthcare providers.	
Outcomes	• Symptoms of depression (continuous or dichotomous) or diagnosis of MDD (using a validated diagnostic interview) • Health-related quality of life • Day-to-day functionality • Lost time at work/school • Impact on lifestyle behaviour (alcohol abuse, smoking, drugs, gambling, etc.) • Suicidality (suicide ideation, attempt or completion) • False-positive result (positive screen in the absence of depressive disorder), overdiagnosis, or overtreatment • Labelling/stigma • Harms of treatment	
Timing	Published from May 2012 to the date the search is run	
Study design	Randomized controlled trials (RCTs)* including cluster-controlled trials *Trials of screening in which patient eligibility is determined and then patients are enrolled prior to randomization (i.e., to screening or to no screening). Similar depression management and treatment resources are provided to patients in the screening arm of the trial who were identified as depressed as well as patients in either the screening or non-screening arms of the trial who were identified as depressed via other methods (e.g. unaided clinician diagnosis, patient report) [40]	- RCTs where patient eligibility is determined and patients are enrolled after randomization - Non-RCTs, controlled before-after, interrupted times series, cohort studies, case-control studies, cross-sectional studies, case series, case reports, and other publication types (editorials, commentaries, notes, letter, opinions)
Setting	Primary care or other non-mental health clinic settings, including specialty clinics such as rheumatology, obstetrics, and gynaecology.	Studies conducted in mental health or psychiatric settings are excluded.
Language	English and French

**Table 2:** Criteria for inclusion and exclusion of studies in the pregnant and postpartum review

	Inclusion criteria	Exclusion criteria
Population	Key question 1: patients during pregnancy and up to 1 year postpartum of any age Key question 1a: patients during pregnancy and up to 1 year postpartum selected for screening because they have characteristics that may suggest an elevated risk of depression* *characteristics as defined in primary studies (e.g. trauma early in life, a family history of depression)	- If > 20% of women have a recent history of depression, have a current diagnosis, or are receiving treatment for depression or other mental disorders (unless results are provided separately from the population of interest) - Women with a history of depression during pregnancy or the postpartum period - Women seeking services due to symptoms of mental disorders - Women receiving assessment or care in psychiatric or mental health settings
Intervention	Interventions that use a single question, small sets of questions, or a screening questionnaire (validated or non-validated) with a pre-defined cut-off score to identify patients who may have depression, but who have not reported their symptoms to healthcare providers or who have otherwise not been identified as possibly depressed by healthcare providers.	Interventions that, in addition to screening, include depression care referral or treatment options that are not available to patients identified as depressed in the no screening trial arm
Comparator	No depression screening Patients in comparator trial arms may be administered depression symptom questionnaires for the purpose of baseline or outcome assessments as long as scores are not provided to the patients or healthcare providers.	
Outcomes	Mental health outcomes • Symptoms of depression (continuous or dichotomous) or diagnosis of MDD (using a validated diagnostic interview) • Health-related quality of life (validated tools) • Suicidality (suicide ideation, attempt, or completion) • False-positive screens (positive screens in the absence of depressive disorder), overdiagnosis, or overtreatment • Labelling/stigma • Harms of treatment Parenting outcomes • Relationship with partner and other supports • Reported/observed capacity to parent (attachment, responsiveness to infant, positive regard of infant/fetus) • Mother-child interactions including mutual touching, smiling, vocalizations, and impact on other children Infant outcomes • Infant health and development (i.e., developmental delay; failure to thrive) cognitive, emotional, motor and neural functioning and development • Infant responsiveness	
Timing	Published from database inception to the date the search is run	
Study design	Randomized controlled trials (RCTs)* including cluster-controlled trials *Trials of screening in which patient eligibility is determined and then patients are enrolled prior to randomization (i.e., to screening or to no screening). Similar depression management and treatment resources are provided to patients in the screening arm of the trial who were identified as depressed as well as patients in either the screening or non-screening arms of the trial who were identified as depressed via other methods (e.g. unaided clinician diagnosis, patient report) [40]	RCTs where patient eligibility is determined, and patients are enrolled after randomization Non-RCTs, controlled before-after, interrupted times series, cohort studies, case-control studies, cross-sectional studies, case series, case reports, and other publication types (editorials, commentaries, notes, letter, opinions)
Setting	Primary care or other non-mental health clinic settings, including specialty clinics such as obstetrical, maternal-fetal medicine, and paediatric clinics	Studies conducted in mental health or psychiatric settings
Language	English and French	

### Data sources and search for studies

Search strategies for each population have been developed using a resource librarian and tested through an iterative process by an experienced medical information specialist in consultation with the review team. Using the OVID platform, we will search Ovid MEDLINE®, Ovid MEDLINE® Epub Ahead of Print, In-Process & Other Non-Indexed Citations, Embase Classic + Embase, PsycINFO, and CINAHL. We will also search the Cochrane Library on Wiley. When possible, animal-only and opinion pieces will be removed from the results. There will be no language restriction in either search. A randomized controlled trial (RCT) filter based on the Cochrane Highly Sensitive Search Strategy, sensitivity- and precision-maximizing version (2008 revision), will be used. Vocabulary and syntax will be adjusted across databases. The final searches have been peer-reviewed using the PRESS 2015 guideline [49].

#### General adult review

The general adult review will update the SR used by the CTFPHC for their previous guideline recommendation [41, 42]. A comprehensive search strategy was developed using the previous SR search strategy as guidance. The search will start from the last search date of the previous review (May 2012). Strategies will utilize a combination of controlled vocabulary (e.g. “Depressive Disorder”, “Mass Screening”, “Adult”) and keywords (e.g. “depression”, “screening”, “adults”) (see Additional file 4 for the OVID multifile search).

#### Pregnant and postpartum review

There will be no date restriction in the search. Strategies use a combination of controlled vocabulary (e.g. “Depressive Disorder”, “Mass Screening”, “Pregnancy Complications”) and keywords (e.g. “depression”, “screening”, “pregnancy”) (see Additional file 5 for the OVID multifile search).

For both SRs, we will search the grey literature for unpublished documents (e.g. reports, theses, governmental publications) following the Canadian Agency for Drugs and Technologies in Health (CADTH) Grey Matters checklist. The CADTH checklist includes national and international health technology assessment agencies, clinical practice guideline organizations, clinical trials registries, Canadian health prevalence and incidence databases, statistics, search engines, and additional databases. The clinical trial registries listed within the checklist include ClinicalTrials.gov, WHO International Clinical Trials Registry Platform, ISRCTN Registry, CenterWatch, and Clinical Trials Registry India. We will supplement the CADTH checklist by searching the websites of the following organizations: the College of Family Physicians, the American College of Physicians, the American Academy of Family Physicians, the Canadian Nurses Association, the American Nurses Association, the Canadian Psychiatric Association, the Centre for Addiction and Mental Health, the Anxiety and Depression Association of America, and the American Psychological Association. Additionally, for the pregnant and postpartum review, we will search the following websites: the Society of Obstetricians and Gynaecologists of Canada, the American College of Obstetricians and Gynecologists, the Royal College of Obstetricians and Gynaecologists, and the Royal Australian and New Zealand College of Obstetricians and Gynaecologists, and the Canadian Association of Midwives.

Grey literature searching will be confined to what can be accomplished within 1 week of searching by one person, for pragmatism, and will be restricted to English and French language documents.

### Screening and data extraction

Search strategies will be run separately for each population. Within each population, duplicates across searches will be identified and removed using Reference Manager [50]. The remaining unique articles will be uploaded into an online SR managing software (DistillerSR©) [51] in two separate projects. For each population, screening will be done in two stages. The first stage is a broad screening of the titles and abstracts. For those deemed potentially relevant based on title and abstract, a more focused screening of the full texts will be evaluated against the population, intervention, and comparison of interest. Draft screening forms can be found in Additional file 6. Title and abstract screening will consist of two reviewers screening for relevance. We will use a liberal accelerated method in which a second reviewer will verify those records deemed not relevant by the first reviewer [52]. As these are done concurrently and randomly, each reviewer will not necessarily know if the reference has already been considered irrelevant by the other reviewer. Conflict resolution will not be done at this stage. At the full-text reviewing stage, two reviewers will independently assess the article for relevancy based on all eligibility criteria. Conflicts will be resolved by consensus or a third team member. Reports that are co-publications or multiple reports of the same study will be identified at full-text review and labelled as such. Only English and French articles will be included at the full-text stage; all other languages will be excluded and labelled as “other language”. A pilot-testing phase among reviewers will be implemented on a sample of articles prior to commencement of full screening at both title and abstract level (50 records) and full-text level (25 records). Articles not available electronically will be ordered via interlibrary loan. If the article is not received within 30 days, it will be excluded and the reason for exclusion will be labelled as “full-text not available”.

For feasibility, conference abstracts have been removed from the search results in Embase and Cochrane, a feature only available in these two databases. If abstracts remain from other databases, reports in abstract form will be noted as such and excluded. A list of potentially relevant studies available only in abstract form will be made available as part of the list of excluded studies. A list of grey literature sources, including registries for on-going or completed studies, will be provided for each question. Working group members and clinical experts will be contacted and invited to submit research reports for consideration. We will consult with the working group members and clinical experts for missing studies. In the cases where a relevant secondary evidence report (e.g. evidence-based clinical practice guidelines, SRs, and meta-analyses) is found, the reference list will be reviewed. Using Robinson et al. [53] as guidance, a SR would need to meet the following criteria to be considered systematic; otherwise, it would be considered a narrative review: (i) at least one database was searched; (ii) it reports selection criteria; (iii) quality appraisal of included studies is reported; and (iv) it provides a list and synthesis of included studies. For full-text screening, where study eligibility is unclear, authors will be contacted by email twice, 2 weeks apart, for additional information. If no response is received, the article will be excluded and will be included in the list of excluded studies as “unclear” for the related question.

Standardized data extraction forms will be developed a priori in DistillerSR and pilot tested, independently in duplicate, on a sample of studies, with this number dependent on the number of included studies (typically 5). Draft items for data extraction are available in Additional file 7. Full data abstraction will be completed by one reviewer and verified by a second reviewer. Disagreements will be resolved by consensus or third-party adjudication if consensus cannot be reached. To facilitate consistent presentation and synthesis of the results across studies, we will convert data (e.g. standard error to standard deviation or 95% confidence intervals). All formats of continuous outcome data will be extracted whether reported as post-intervention or change from baseline. As done previously [54], where needed, a conservative value for a correlation coefficient of 0.25 will be used to impute standard deviations for means used in change from baseline calculations. Authors will be contacted by email twice over 2 weeks, if any information is missing, or unclear. If no response is received, the outcome will not be included in the synthesis, but will be discussed in the corresponding outcome results section.

### Risk of bias assessment

We will use the Cochrane risk of bias (ROB) tool to assess the ROB of included trials [55]. This will be performed by one reviewer with verification completed by a second reviewer. Disagreements will be resolved by consensus or third-party adjudication. Some domains in the Cochrane ROB are outcome-specific (e.g. blinding of outcome assessors) and will be assessed at the outcome level. The overall ROB for the body of evidence will involve a judgement of the relative importance of domains, guided by known empirical evidence of bias, the likely direction of bias, and the likely magnitude of bias [55]. We will follow the GRADE guidance for determining the extent of the ROB for the body of evidence [56]. For outcome and analysis reporting bias, we will use the methods outlined in the Agency for Healthcare Research and Quality guidance to determine ROB for that domain [57]. When assessing cluster randomized trials, we will assess for the possibility of recruitment bias in the “other bias” domain of the Cochrane ROB [58].

### Data synthesis and statistical analysis

Study characteristics, including country of conduct, author(s), date of publication, number of included participants in each group, location of intervention (e.g. general physicians clinic), and funding, will be summarized narratively and presented in summary tables. Where possible, relative and absolute effects with 95% confidence intervals will be calculated to facilitate presentation of outcome data according to the GRADE summary of findings and evidence profile tables adopted by the CTFPHC. For example, risk ratios and risk differences will be ideally used to report effects for binary data. GRADE guidance will be used for presenting continuous data [59]. Where possible, the number needed to treat/harm will be calculated.

#### Meta-analysis

We will determine whether clinical and methodological heterogeneity exists among studies, prior to performing a meta-analysis. If it is determined to be appropriate, based on clinical similarity between studies and that the body of evidence is not at high risk of bias, data will be meta-analysed using random effects models for effect measures such as risk ratios and risk differences. If it is determined that meta-analysis is not appropriate, the range of effects will be presented. For time-to-event data, the hazard ratio will be pooled using the generic inverse variance method.

Unit of analysis errors can occur in studies that employ a cluster design (e.g. a clinical practice) and yet are analysed at the individual level (e.g. patients), potentially leading to artificially precise results and contributing more weight than would be appropriate in a meta-analysis [60]. If empirically derived intra-cluster correlation coefficients are available, we will adjust the analysis to address these errors [61]. For multiple events that may occur in one person (e.g. suicide attempt), we will assume each event represents a unique individual, unless data are presented as events per individual. If we were to encounter a study where there is reason for concern that many events are recorded in a small percentage of research participants, the impact of this study could be evaluated in a sensitivity analysis.

#### Sparse binary data and studies with zero events

When studies report rare events, a synthesis will be done narratively. For those outcomes (e.g. suicide completion) where at least one intervention group contains zero events, only the risk difference (RD) will be used. For calculating the RD, we will use the median baseline risk for the control group in the included studies, although we may additionally perform sensitivity analyses using differing baseline risks if thought suitable.

#### Statistical heterogeneity

The Cochran’s *Q* and *I*^2^ statistic will be used to assess the statistical heterogeneity of effect estimates among included studies. For the interpretation of *I*^2^, a rough guide of low (0–25%), moderate (25–50%), substantial (50–75%), and considerable (≥ 75%) will be used [62, 63]. Should considerable statistical heterogeneity exist, we will present all studies in a forest plot, but will not provide the pooled estimate. When the body of evidence is statistically heterogeneous, we will conduct subgroup, sensitivity analysis, and/or meta-regression analyses, where the optimal approach for each variable will be determined once we see how data are reported in studies. We will follow previously published guidance for meta-regression [64]. Meta-regression will be based on random effects models to allow for residual unexplained heterogeneity. In consideration of the low power of the *Q* statistic, when the number of studies is small and the possibility of detecting unimportant heterogeneity when the number of studies is large, a *p* value < 0.10 will characterize statistical significance [64]. When the sizes of the included studies are moderate or large, there should be at least 10 studies for a continuous study-level variable. For a categorical subgroup variable, each subgroup should have a minimum of four studies. These numbers serve as the lower bounds for considering meta-regression [64]. When included studies are mostly small in size, univariate meta-regression will be used when an insufficient number of studies are available to conduct multivariable analyses. We will not pool outcomes if there is an *I*^2^ of > 75%. We will use the *p* value from the chi-square test as support to interpret the strength of evidence for heterogeneity.

#### Subgroup analysis

The following subgroup analyses are planned in Table 3.

**Table 3:** Planned subgroup analysis

Key questions	Both populations	General adult	Pregnancy and postpartum
1 and 1a	▪ Socioeconomic status (e.g. income, level of education, as assessed by study authors) ▪ Race/ethnicity (will be determined post hoc, depending on populations encountered in studies) ▪ Geographical location (e.g. rural vs urban settings, country/region) ▪ Validated vs non-validated tools	▪ Gender/sex	▪ Timing period and frequency of screening (e.g. prenatal, immediate postpartum)
1 only	▪ Age groups (e.g. < 25 years of age) ▪ Immigrant status		▪ Support status (e.g. single mother with no family support vs other) ▪ Partum status (e.g. first child vs later)
1a only	▪ Risk factors for depression (to be determined post hoc, depending on the combination of risk factors as reported in studies).		

#### Sensitivity analyses

Sensitivity analyses may be undertaken to restrict analyses to those studies assessed as being of low ROB, based on the overall judgement, and may also be performed to address any decisions made regarding handling of data or to explore statistical heterogeneity. A sensitivity analysis may also be performed on the timing of publication, based on cut-offs as determined by literature and any other design-specific issues we may come across.

#### Small study effects

If there is a minimum of 10 studies in any meta-analysis, we will assess for small study effects using a combination of graphical aids (e.g. funnel plot) and/or statistical tests (e.g. Egger regression test, Hedges-Olkin) [63]. Funnel plot asymmetry can be used to identify potential bias, as well as signal exaggeration of treatment effects in small studies [65].

#### Software

The Cochrane Review Manager software version 5.3 will be used to calculate effect estimates and conduct meta-analyses [66]. For all analyses not possible in RevMan v5.3, we will use Comprehensive Meta-Analysis v3.

#### Grading the quality of evidence and interpretation

We will assess the quality of evidence for individual comparisons and outcomes using the GRADE approach. GRADE tables will be prepared for each of the critical and important outcomes using the GRADE framework to assess each domain (i.e., risk of bias, imprecision, inconsistency, indirectness, and publication bias) [44, 45]. This will be performed by one reviewer. Verification will be completed by a second reviewer. Disagreements will be resolved by consensus or third-party adjudication.

## Discussion

We will publish the results of these reviews in the ‘Canadian Task Force on Preventive Health Care Evidence Reviews’ series. The findings from both reviews will build the foundation for future research and highlight the implications for primary care practice, and the results will be used by the CTFPHC to help develop their guideline recommendations on depression screening in Canada.

## Additional files

Additional file 1: DSM-5 and ICD-10 definition of major depressive episode. (DOCX 14 kb)

Additional file 2: Current recommendations from guideline organizations. (DOCX 33 kb)

Additional file 3: PRISMA-P 2015 checklist. (DOCX 18 kb)

Additional file 4: Search strategy for general adult population. (DOCX 18 kb)

Additional file 5: Search strategy for pregnant and postpartum women. (DOCX 18 kb)

Additional file 6: Draft screening forms. (DOCX 14 kb)

Additional file 7: Draft items for data extraction. (DOCX 14 kb)

Additional file 8: Stakeholder review and feedback. (DOCX 38 kb)

## Abbreviations

AMSTAR 2: Assess the Methodological Quality of Systematic Reviews
CADTH: Canadian Agency for Drugs and Technologies in Health
CANMAT: Canadian Network for Mood and Anxiety Treatments
CI: Confidence interval
CINAHL: Cumulative Index to Nursing and Allied Health Literature
CTFPHC: Canadian Task Force on Preventive Health Care
CVD: Cardiovascular disease
DSM-5: Diagnostic and Statistical Manual of Mental Disorders, Fifth Edition
EPDS: Edinburgh Perinatal/Postpartum Depression Scale
ERSC: Evidence Review and Synthesis Centre
GRADE: Grading of Recommendations Assessment, Development and Evaluation
ICD-10: International Classification of Diseases, 10th Revision
ISRCTN: International Standard Randomised Controlled Trial Number
MDD: Major depressive disorder
MDE: Major depressive episode
PHAC: Public Health Agency of Canada
PICO: Population, Intervention, Comparator, Outcome
PRESS: Peer Review of Electronic Search Strategies
PRISMA: Preferred Reporting Items for Systematic Reviews and Meta-Analyses
PRISMA-P: Preferred Reporting Items for Systematic Reviews and Meta-Analyses Protocols
PROSPERO: International Prospective Registry of Systematic Reviews
RCT: Randomized controlled trial
RD: Risk difference
ROB: Risk of bias
SR: Systematic review
UKNSC: United Kingdom National Screening Committee
USPSTF: US Preventive Services Task Force
