# Supplementary material for: Screening for depression in women during pregnancy or the first year postpartum and in the general adult population: a protocol for two systematic reviews to update a guideline of the Canadian Task Force on Preventive Health Care
Source: Syst Rev. 2019 Jan 19;8:27. doi: 10.1186/s13643-018-0930-3 (PMC6339426; doi:10.1186/s13643-018-0930-3)
Supplement: Supplementary file 1 — DSM-5 and ICD-10 definition of major depressive episode. (DOCX 14 kb) [file 13643_2018_930_MOESM1_ESM.docx]

## **Additional file 1. DSM-5 and ICD-10 definition of major depressive episode**

| **DSM-5^ϯ^ - Major Depressive Episode** | **ICD-10^Ϯ^ - Depressive Episode** |
| --- | --- |
| **Criteria that must be met** | |
| [A] Five or more of the symptoms listed below (i-ix) have been present during the same 2-week period and represent a change from previous functioning. At least one of the symptoms is either (i) or (ii).  [B] The symptoms must cause clinically significant distress or impairment in social, occupational, or other important areas of functioning;  [C] The episode is not attributable to the physiological effects of a substance or to another medical condition.  **Criteria A-C represents a major depressive episode**  [D] The occurrence of the major depressive episode is not better explained by schizoaffective disorder, schizophrenia, schizophreniform disorder, delusional disorder, or other specified and unspecified schizophrenia spectrum and other psychotic disorders;  [E] There has never been a manic episode or a hypomanic episode.  **Criteria A-E represents major depressive disorder** | [1] The minimum duration of the whole episode is about 2 weeks;  At least two of the following should be present:  [a] depressed mood  [b] loss of interest and enjoyment  [c] reduced energy leading to increased fatigability and diminished activity |
| **Additional criteria to be met** | |
| [i] Depressed mood most of the day, nearly every day, as indicated by either subjective report (e.g., feels sad, empty, hopeless) or observation made by others (e.g., appears tearful);  [ii] Markedly diminished interest or pleasure in all, or almost all, activities most of the day, nearly every day (as indicated by either subjective account or observation). | *Criteria [a] and [b] above in criteria that must be met.*  Additionally, at least two to four of the following seven must be met: |
| [iii] Significant weight loss when not dieting or weight gain (e.g., a change of more than 5% of body weight in a month) or decrease or increase in appetite nearly every day. | [i] Diminished appetite |
| [iv] Insomnia or hypersomnia nearly every day. | [ii] Disturbed sleep |
| [v] Psychomotor agitation or retardation nearly every day (observable by others, not merely subjective feelings of restlessness or being slowed down). | *[c] above in criteria that must be met* |
| [vi] Fatigue or loss of energy nearly every day. | *[c] as above in criteria that must be met* |
| [vii] Feelings of worthlessness or excessive or inappropriate guilt (which may be delusional) nearly every day (not merely self-reproach or guilt about being sick). | [iii] Ideas of guilt and unworthiness |
| [viii] Diminished ability to think or concentrate, or indecisiveness, nearly every day (either by subjective account or as observed by others). | [iv] Complaints or evidence of diminished ability to think or concentrate, such as indecisiveness or vacillation |
| [ix] Recurrent thoughts of death (not just fear of dying), recurrent suicidal ideation without a specific plan, or a suicide attempt or a specific plan for committing suicide. | [v] Ideas or acts or self-harm or suicide |
|  | [vi] Bleak and pessimistic views of the future |
|  | [vii] Reduced self-esteem and self-confidence |

ϯ *Diagnostic and Statistical Manual of Mental Disorders, Fifth Edition*

Ϯ “Depressive Episodes” in the *ICD-10 Classification of Mental and Behavioural Disorders. Clinical descriptions and diagnostic guidelines*
