# Supplementary material for: Screening for depression in women during pregnancy or the first year postpartum and in the general adult population: a protocol for two systematic reviews to update a guideline of the Canadian Task Force on Preventive Health Care
Source: Syst Rev. 2019 Jan 19;8:27. doi: 10.1186/s13643-018-0930-3 (PMC6339426; doi:10.1186/s13643-018-0930-3)
Supplement: Supplementary file 2 — Current recommendations from guideline organizations. (DOCX 33 kb) [file 13643_2018_930_MOESM2_ESM.docx]

# Additional file 2. Screening for depression in the pregnant and postpartum population

## Current Practice in Canada

Across Canada, there is a lack of consensus on how and when prenatal and postpartum depression screening should occur. Typically, it varies by each provincially and territorially developed program. Antenatal records provide guidance for care during pregnancy and allow the primary care provider (e.g., physician, nurse, midwife) to record information during appointments as pregnancy progresses (e.g., body weight, labour plans). In most cases, follow-up is typically limited to the time leading up to and including the 6-week postpartum follow-up appointment. For example, the Ontario Perinatal Record 3 covers mental health in the second and third trimester as part of the “discussion topics”, and states that mental health assessment should be an ongoing process and that the screening tools in the Ontario Perinatal Record 4. Resources can be used at any time throughout the pregnancy. The tools include the Generalized Anxiety Disorder Scale, the Patient Health Questionnaire-2, and the Edinburgh Perinatal/Postpartum Depression Scale (EPDS). The Ontario Perinatal Record 5 is specific to the postnatal visit and recommends screening all clients/patients for postpartum depression with the screening tools suggested in the Ontario Perinatal Record 4 [1].

Other provinces have their own versions of these records. The BC Antenatal Record 2 recommends using [2], [3]the EPDS screening tool in all pregnancies between 28-32 weeks and women 6-8 weeks postpartum [4], and the BC Community Postpartum Assessment assesses the emotional status using the Edinburgh Postpartum Depression score [5]. Alberta provides physicians and midwives a prenatal care worksheet, informing users to inquire about general well-being at each prenatal visit, and to assess for postpartum depression using the EPDS screening tool at around 2 months postpartum [6]–[8]. Nova Scotia has a series of three prenatal records [9]. Included within Prenatal Record 1, a woman is asked about current depression/anxiety, Prenatal Record 2 lists postpartum depression as a topic to be discussed, and Prenatal Record 3 allows the physician to list any problems, of which history of depression is one suggested in the companion document [10]. In Nunavut, depression screening is done using the EPDS during the second or third trimester [11].

## Current Recommendations from the CTFPHC and Other Guideline Organizations

*CTFPHC.* In 2013, the CTFPHC published recommendations on screening for depression in adults who are at average risk and those who may be at increased risk of depression [12]. This guideline considered the perinatal and postpartum population as a subgroup who may be at increased risk of depression. The CTFPHC suggested not to routinely screen (very low-quality evidence); this was deemed a weak recommendation [12] and was based primarily on evidence from a 2013 systematic review by Keshavarz et al. [13]. The systematic review did not include any eligible studies showing the benefits or harms of screening.

*Other Guidelines.* The UK National Screening Committee’s (UKNSC) postnatal depression screening recommendation, last updated in 2011, recommended against a screening program [14]. This recommendation was based on evidence that the use of current identification strategies would result in a significant number of false positives. They emphasized the lack of evidence from trials and found insufficient evidence that screening significantly improved health outcomes for mother or baby.

In contrast, the 2015 guideline from the American College of Obstetricians and Gynecologists recommended screening patients at least once during the perinatal period for depression and anxiety symptoms using a standardized, validated tool, despite limited evidence of benefit [15]. This recommendation was made based on a systematic review by Myers et al (2013) [16]. For their key question on the benefits and harms of screening, they included five studies, four RCTs and one quasi-experimental study. Only one trial was considered “good quality”, although the authors had combined screening and treatment in the intervention arm and did not offer the same treatment to the control arm. Two trials were considered “fair” and one was “poor.” The quasi-experimental study was rated “poor quality.”

In 2016, the U.S. Preventive Services Task Force (USPSTF) updated their guideline and recommended screening for depression in the general adult population in primary care settings, when adequate systems were in place to ensure accurate diagnosis, effective treatment, and appropriate follow-up. This recommendation included pregnant and postpartum women [17]. The recommendation was based primarily on a systematic review by O’Connor et al (2016) that focused on pregnant and postpartum women [18]. The authors included six trials that they described as trials that examined the benefits of screening and found no evidence on the harms of screening. One trial was considered “good quality” while the others were rated as “fair quality.”

Thombs et al (2017) compared the recommendations from the CTFPHC, the USPSTF, and the UKNSC to evaluate the consistency and sources of divergence between these three guidelines. Neither the CTFPHC or UKNSC recommended screening in contrast to the USPFTF recommendation for screening on results that combined screening with treatment [19]. They only considered RCTs that compared screening to no screening in studies where patients were randomized prior to screening and where patients with depression in both screened and non-screened groups had access to similar depression care options. None of these guidelines identified direct evidence that screening improves health outcomes. Regardless of this, the USPSTF relied on indirect evidence (e.g., studies of test accuracy, treatment of screen-detected symptomatic patients) for their recommendation for screening. They relied on trials of depression care management programs, which used screening tools to establish trial eligibility prior to randomization, including trials of collaborative care treatment versus usual care treatment among people already diagnosed with depression as a condition of being in the trial. They also included studies that combined screening and treatment and did not offer the same treatment resources to the control arm, therefore, screening and treatment effects could not be differentiated. In addition, the USPSTF described harms as small to negligible, did not mention overdiagnosis or overtreatment, and did not examine the cost and resource considerations of screening, all of which were emphasized in the CTFPHC and UKNSC recommendations.

# Screening for depression in the general adult population

## Current Recommendations from the CTFPHC and Other Guideline Organizations

*CTFPHC*: In 2013, the CTFPHC published recommendations on screening for depression in adults who are at average risk and those who may be at increased risk of depression. The systematic review did not identify any trials that demonstrated direct evidence of the benefits or harms of screening [13]. The recommendations for the general adult population was to not routinely screen, this was based on very-low quality evidence and was classified as a “weak recommendation” [12].

*Other Guidelines*: The UK National Screening Committee (2015) did not recommend screening the general population for depression. This recommendation was guided by a report by Pittam and Allaby (2014) [20], in particular noting that questionnaire-based tests used to identify people who are at risk of depression are not sufficiently accurate when used in the general population, and many people would be falsely identified as having depression. In addition, although screening might help identify some people who are at high risk of developing depression, there was no clear evidence that treatment would prevent those with mild depression going on to develop severe depression.

The 2016 U.S. Preventive Services Task Force (USPSTF) updated guideline recommended screening for depression in the general adult population with adequate systems in place to ensure accurate diagnosis, effective treatment, and appropriate follow-up [17]. They stated that the direct evidence of benefits of screening was weak, but the totality of the evidence assists the benefits of screening and considered this net benefit as moderate to substantial. The recommendation was made based primarily on a systematic review by O’Connor et al (2016) [21]. The review authors included nine trials that examined the benefits of screening and found no evidence on the harms of screening. Only two trials were considered “good quality” while the others were rated as “fair quality.”

Thombs et al (2017) compared the recommendations from the CTFPHC, the USPSTF, and the UKNSC to evaluate the consistency and sources of divergence between these three guidelines. Neither the CTFPHC or UKNSC recommended screening in contrast to the USPFTF recommendation for screening on results that combined screening with treatment [19]. The guidelines only considered RCTs that compared screening to no screening in studies where patients were randomized prior to screening and where patients with depression in both screened and non-screened groups had access to similar depression care options. None of these guidelines identified direct evidence that screening improves health outcomes. Regardless of this, the USPSTF relied on indirect evidence (e.g., studies of test accuracy, treatment of screen-detected symptomatic patients) for their recommendation for screening. They relied on trials of depression care management programs, which used screening tools to establish trial eligibility prior to randomization, including trials of collaborative care treatment versus usual care treatment among people already diagnosed with depression as a condition of being in the trial. They also included studies that combined screening and treatment and did not offer the same treatment resources to the control arm, therefore, screening and treatment effects could not be differentiated. In addition, the USPSTF described harms as small to negligible, did not mention overdiagnosis or overtreatment, and did not examine the cost and resource considerations of screening, all of which were emphasized in the CTFPHC and UKNSC recommendations.

# References

[1] Ministry of Health and Long-Term Care, “Ontario Perinatal Record 1-5.” Oct-2017.

[2] Perinatal Services BC, “Postpartum and Newborn Care Summary Checklist for Primary Care Providers.” Oct-2016.

[3] Cox, JL, Holden, JM, and Sagovsky, R, “Detection of postnatal depression. Development of the 10-item Edinburgh Postnatal Depression Scale. [document used for Perinatal Services BC - Edinburgh Perinatal/Postnatal Depression Scale (EPDS)].” 1987.

[4] Perinatal Services BC, “Antenatal Record 1 and 2.” Oct-2011.

[5] Perinatal Services BC, “British Columbia Community Postpartum Assessment.” Jan-2011.

[6] Alberta Prenatal Health Program, “Alberta Prenatal Record.” .

[7] Maternal Newborn Child & Youth Strategic Clinical Network, “Alberta Pregnancy Pathways.” Sep-2017.

[8] MyHealthAlberta.ca, “Health Information and Tools - Postpartum Depression,” May-2017. [Online]. Available: https://myhealth.alberta.ca/Health/pages/conditions.aspx?hwId=tn9653#tn9660.

[9] Reproductive Care Program of Nova Scotia, “Nova Scotia Prenatal Record 1-3.” [Online]. Available: http://rcp.nshealth.ca/chart-prenatal-forms/nova-scotia-prenatal-record. [Accessed: 04-Apr-2018].

[10] Reproductive Care Program of Nova Scotia, “Nova Scotia Prenatal Record Companion Document.” [Online]. Available: http://rcp.nshealth.ca/publications/nova-scotia-prenatal-record-companion-document.

[11] Nunavut Department of Health, “Guidelines for Completing Prenatal Record.” Jan-2016.

[12] Canadian Task Force on Preventive Health Care *et al.*, “Recommendations on screening for depression in adults,” *CMAJ*, vol. 185, no. 9, pp. 775–782, 11 2013.

[13] H. Keshavarz *et al.*, “Screening for depression: a systematic review and meta-analysis,” *CMAJ Open*, vol. 1, no. 4, pp. E159-167, Oct. 2013.

[14] “The UK NSC recommendation on Postnatal depression screening in pregnancy,” *Current UK NSC recommendations*. [Online]. Available: https://legacyscreening.phe.org.uk/postnataldepression. [Accessed: 03-Apr-2018].

[15] American College of Obstetricians and Gynecologists, “Screening for perinatal depression. Committee Opinion No. 630,” *Obstet Gynecol*, vol. 125, pp. 1268–1271, 2015.

[16] E. R. Myers *et al.*, *Efficacy and Safety of Screening for Postpartum Depression*. Rockville (MD): Agency for Healthcare Research and Quality (US), 2013.

[17] A. L. Siu *et al.*, “Screening for Depression in Adults: US Preventive Services Task Force Recommendation Statement,” *JAMA*, vol. 315, no. 4, pp. 380–387, Jan. 2016.

[18] E. O’Connor, R. C. Rossom, M. Henninger, H. C. Groom, and B. U. Burda, “Primary Care Screening for and Treatment of Depression in Pregnant and Postpartum Women: Evidence Report and Systematic Review for the US Preventive Services Task Force,” *JAMA*, vol. 315, no. 4, pp. 388–406, Jan. 2016.

[19] B. D. Thombs *et al.*, “Consistency and sources of divergence in recommendations on screening with questionnaires for presently experienced health problems or symptoms: a comparison of recommendations from the Canadian Task Force on Preventive Health Care, UK National Screening Committee, and US Preventive Services Task Force,” *BMC Med*, vol. 15, no. 1, p. 150, 09 2017.

[20] G. Pittam and M. Allaby, “Appraisal of Screening for Depression. A report for the UK National Screening Committee,” Aug. 2014.

[21] E. O’Connor *et al.*, *Screening for Depression in Adults: An Updated Systematic Evidence Review for the U.S. Preventive Services Task Force*. Rockville (MD): Agency for Healthcare Research and Quality (US), 2016.
