# Supplementary material for: Screening for depression in women during pregnancy or the first year postpartum and in the general adult population: a protocol for two systematic reviews to update a guideline of the Canadian Task Force on Preventive Health Care
Source: Syst Rev. 2019 Jan 19;8:27. doi: 10.1186/s13643-018-0930-3 (PMC6339426; doi:10.1186/s13643-018-0930-3)
Supplement: Supplementary file 4 — Search strategy for general adult population. (DOCX 18 kb) [file 13643_2018_930_MOESM4_ESM.docx]

# Additional file 4. Search strategy for general adult population (OVID multi-file)

Depression Screening – Adults

2018 Aug 14

Ovid Multifile

Database: Embase Classic+Embase <1947 to 2018 August 13>, Ovid MEDLINE(R) ALL <1946 to August 13, 2018>, PsycINFO <1806 to August Week 1 2018>

Search Strategy:

--------------------------------------------------------------------------------

1 exp Depressive Disorder/ (500056)

2 Depression/ (439468)

3 depress*.tw,kf. (1249935)

4 dysthym*.tw,kf. (10782)

5 blues.tw,kf. (4486)

6 melanchol*.tw,kf. (11557)

7 MDD.tw,kf. (34228)

8 or/1-7 [DEPRESSION] (1436847)

9 Mass Screening/ (149657)

10 (screen* or detect*).tw,kf. (6025611)

11 (identif* or recogni*).ti. (782823)

12 ((early or earlier or earliest) adj5 (identif* or recogni*)).tw,kf. (159075)

13 (case finding? or casefinding?).tw,kf. (11892)

14 or/9-13 [SCREENING] (6782413)

15 8 and 14 [SCREENING FOR DEPRESSION] (140618)

16 (controlled clinical trial or randomized controlled trial or pragmatic clinical trial).pt. (554825)

17 clinical trials as topic.sh. (184495)

18 exp Randomized Controlled Trials as Topic/ (240765)

19 (randomi#ation? or randomi#ed or randomly or RCT$1 or placebo*).tw,kf. (2164491)

20 ((singl* or doubl* or trebl* or tripl*) adj (mask* or blind* or dumm*)).tw,kf. (394881)

21 trial.ti. (455346)

22 or/16-21 (2740269)

23 15 and 22 [SCREENING FOR DEPRESSION - RCTS] (14480)

24 exp Adult/ (13708110)

25 (adult or adults or adulthood).tw,kf. (2933073)

26 (man or men or woman or women).tw,kf. (3896526)

27 middle-age?.tw,kf. (115191)

28 age?.tw,kf. (6625889)

29 (elderly or geriatric* or gerontolog* or old-age? or senior?).tw,kf. (853046)

30 (older adj2 (female? or male? or patient? or person? or people? or population?)).tw,kf. (272555)

31 or/24-30 [ADULTS] (19902501)

32 23 and 31 [SCREENING FOR DEPRESSION - ADULTS - RCTS] (10157)

33 exp Child/ not (exp Adult/ and exp Child/) (3111046)

34 exp Child/ not (Adolescent/ and exp Child/) (2860576)

35 exp Infant/ not (exp Adult/ and exp Infant/) (1623339)

36 exp Infant/ not (Adolescent/ and exp infant/) (1637771)

37 or/33-36 (4058903)

38 32 not 37 [CHILD-/INFANT-ONLY REMOVED] (9676)

39 exp Animals/ not (exp Animals/ and Humans/) (16785381)

40 38 not 39 [ANIMAL-ONLY REMOVED] (6821)

41 (comment or editorial or interview or news or newspaper article).pt. (1816246)

42 (letter not (letter and randomized controlled trial)).pt. (1968033)

43 40 not (41 or 42) [OPINION PIECES REMOVED] (6815)

44 limit 43 to yr="2012-current" (2429)

45 44 use medall [MEDLINE RECORDS] (1690)

46 exp Depressive Disorder/ (500056)

47 depress*.tw,kw. (1256850)

48 dysthym*.tw,kw. (10863)

49 blues.tw,kw. (4502)

50 melanchol*.tw,kw. (11638)

51 MDD.tw,kw. (34353)

52 or/46-51 [GENERAL DEPRESSION] (1419516)

53 screening/ (278857)

54 mass screening/ (149657)

55 screening test/ (67501)

56 (screen* or detect*).tw,kw. (6034447)

57 (identif* or recogni*).ti. (782823)

58 ((early or earlier or earliest) adj5 (identif* or recogni*)).tw,kw. (159175)

59 (case finding? or casefinding?).tw,kw. (12002)

60 or/53-59 [GENERAL SCREENING] (6817028)

61 depression assessment/ (843)

62 52 and 60 (141624)

63 61 or 62 [DEPRESSION & SCREENING/ASSESSMENT] (142284)

64 randomized controlled trial/ or controlled clinical trial/ (1219653)

65 exp "clinical trial (topic)"/ (231597)

66 (randomi#ation? or randomi#ed or randomly or RCT$1 or placebo*).tw,kw. (2166401)

67 ((singl* or doubl* or trebl* or tripl*) adj (mask* or blind* or dumm*)).tw,kw. (395018)

68 trial.ti. (455346)

69 or/64-68 (2890315)

70 63 and 69 [RCTs - DEPRESSION & SCREENING/ASSESSMENT] (15596)

71 exp adult/ (13708110)

72 (adult or adults or adulthood).tw,kw. (2938333)

73 (man or men or woman or women).tw,kw. (3897751)

74 middle-age?.tw,kw. (115575)

75 age?.tw,kf. (6625889)

76 (elderly or geriatric* or gerontolog* or old-age? or senior?).tw,kw. (862575)

77 (older adj2 (age? or female? or male? or patient? or person? or people? or population?)).tw,kw. (410390)

78 or/71-77 [ADULT POPULATION] (19905908)

79 70 and 78 [SCREENING FOR DEPRESSION - ADULT POPULATION - RCTs] (10985)

80 exp child/ not (exp adult/ and exp child/) (3111046)

81 exp adolescent/ not (exp adult/ and exp adolescent/) (1095510)

82 fetus/ not (exp adult/ and fetus/) (234148)

83 or/80-82 (3716421)

84 79 not 83 [UNDER-18 POPULATION ONLY REMOVED] (10510)

85 exp animal experimentation/ or exp animal model/ or exp animal experiment/ or nonhuman/ or exp vertebrate/ (46719203)

86 exp human/ or exp human experimentation/ or exp human experiment/ (36484992)

87 85 not 86 (10235912)

88 84 not 87 [ANIMAL-ONLY REMOVED] (10357)

89 editorial.pt. (1004022)

90 letter.pt. not (letter.pt. and randomized controlled trial/) (1963354)

91 88 not (89 or 90) [OPINION PIECES REMOVED] (10349)

92 conference abstract.pt. (3109222)

93 91 not 92 [CONFERENCE ABSTRACTS REMOVED] (8997)

94 limit 93 to yr="2012-CURRENT" (4105)

95 94 use emczd [EMBASE RECORDS] (1933)

96 "Depression (Emotion)"/ (127852)

97 exp Major Depression/ (170646)

98 depress*.tw. (1245207)

99 dysthym*.tw. (10769)

100 blues.tw. (4479)

101 melanchol*.tw. (11518)

102 MDD.tw. (34141)

103 or/96-102 [GENERAL DEPRESSION] (1289710)

104 Screening/ (278857)

105 exp Screening Tests/ (24372)

106 exp Health Screening/ (217092)

107 (screen* or detect*).tw. (6019590)

108 (identif* or recogni*).ti. (782823)

109 ((early or earlier or earliest) adj5 (identif* or recogni*)).tw. (159003)

110 (case finding? or casefinding?).tw. (11839)

111 or/104-110 [GENERAL SCREENING] (6848056)

112 103 and 111 [SCREENING FOR DEPRESSION] (132921)

113 Clinical Trials/ (83742)

114 (randomi#ation? or randomi#ed or randomly or RCT$1 or placebo*).tw. (2162657)

115 ((singl* or doubl* or trebl* or tripl*) adj (mask* or blind* or dumm*)).tw. (394794)

116 trial.ti. (455346)

117 or/113-116 (2411603)

118 112 and 117 [RCTs - SCREENING FOR DEPRESSION] (12986)

119 (adult or adults or adulthood).tw. (2928391)

120 (man or men or woman or women).tw. (3893856)

121 middle-age?.tw. (114710)

122 age?.tw. (6614958)

123 Geriatric Patients/ (12779)

124 (elderly or geriatric* or gerontolog* or old-age? or senior?).tw. (834845)

125 (older adj2 (age? or female? or male? or patient? or person? or people? or population?)).tw. (409004)

126 or/119-125 [ADULT POPULATION] (11466984)

127 118 and 126 (6651)

128 limit 118 to "300 adulthood <age 18 yrs and older>" [Limit not valid in Embase,Ovid MEDLINE(R),Ovid MEDLINE(R) Daily Update,Ovid MEDLINE(R) In-Process,Ovid MEDLINE(R) Publisher; records were retained] (12364)

129 127 or 128 [SCREENING FOR DEPRESSION - ADULTS] (12616)

130 exp Animals/ not (exp Animals/ and Humans/) (16785381)

131 129 not 130 [ANIMAL-ONLY REMOVED] (8590)

132 limit 131 to yr="2012-current" (3281)

133 132 use medall,emczd (2413)

134 132 not 133 [PSYCINFO RECORDS] (868)

135 45 or 95 or 134 [ALL DATABASES] (4491)

136 remove duplicates from 135 (2690) [TOTAL UNIQUE RECORDS]

137 136 use medall [MEDLINE UNIQUE RECORDS] (1671)

138 136 use emczd [EMBASE UNIQUE RECORDS] (763)

139 136 not (137 or 138) [PSYCINFO UNIQUE RECORDS] (256)

***************************
