# Supplementary material for: Screening for depression in women during pregnancy or the first year postpartum and in the general adult population: a protocol for two systematic reviews to update a guideline of the Canadian Task Force on Preventive Health Care
Source: Syst Rev. 2019 Jan 19;8:27. doi: 10.1186/s13643-018-0930-3 (PMC6339426; doi:10.1186/s13643-018-0930-3)
Supplement: Supplementary file 5 — Search strategy for pregnant and postpartum women. (DOCX 18 kb) [file 13643_2018_930_MOESM5_ESM.docx]

## **Additional File 5. Search strategy for pregnant and postpartum women**

Depression – Screening – Pregnancy/Postpartum Population

2018 Aug 14

Ovid Multifile

Database: Embase Classic+Embase <1947 to 2018 August 13>, Ovid MEDLINE(R) ALL <1946 to August 13, 2018>, PsycINFO <1806 to August Week 1 2018>

Search Strategy:

--------------------------------------------------------------------------------

1 exp Depressive Disorder/ (500056)

2 Depression/ (439468)

3 depress*.tw,kf. (1249935)

4 dysthym*.tw,kf. (10782)

5 blues.tw,kf. (4486)

6 melanchol*.tw,kf. (11557)

7 MDD.tw,kf. (34228)

8 PND.tw,kf. (11098)

9 PPD.tw,kf. (21914)

10 or/1-9 [GENERAL DEPRESSION] (1465334)

11 Mass Screening/ (149657)

12 (screen* or detect*).tw,kf. (6025611)

13 (identif* or recogni*).ti. (782823)

14 ((early or earlier or earliest) adj5 (identif* or recogni*)).tw,kf. (159075)

15 (case finding? or casefinding?).tw,kf. (11892)

16 or/11-15 [GENERAL SCREENING] (6782413)

17 10 and 16 (146065)

18 (controlled clinical trial or randomized controlled trial or pragmatic clinical trial).pt. (554825)

19 clinical trials as topic.sh. (184495)

20 exp Randomized Controlled Trials as Topic/ (240765)

21 (randomi#ation? or randomi#ed or randomly or RCT$1 or placebo*).tw,kf. (2164491)

22 ((singl* or doubl* or trebl* or tripl*) adj (mask* or blind* or dumm*)).tw,kf. (394881)

23 trial.ti. (455346)

24 or/18-23 (2740269)

25 17 and 24 [RCTs - DEPRESSION & SCREENING] (14784)

26 exp Pregnancy/ (1594897)

27 exp Pregnancy Complications/ (525880)

28 Pregnant Women/ (58648)

29 exp Pregnancy Trimesters/ (769880)

30 pregnan*.tw,kf. (1131668)

31 Maternal Health Services/ (13097)

32 Peripartum Period/ (23397)

33 exp Perinatal Care/ (60040)

34 Prenatal Care/ (60823)

35 exp Postpartum Period/ (119836)

36 (prenatal* or pre-natal* or antenatal* or ante natal* or antepartum or ante partum or perinatal* or peri-natal* or peripartum or peri-partum or postnatal* or post-natal* or postpartum or post partum or puerperal or puerperium).tw,kf. (767300)

37 Maternal Health/ (13465)

38 (maternal* or maternit*).tw,kf. (613210)

39 (expectant mother* or "new mother" or "new mothers" or "mother-to-be" or "mothers-to-be").tw,kf. (11760)

40 or/26-39 [PREGNANCY/ANTENATAL/POSTNATAL PERIOD] (2611849)

41 25 and 40 [RCTs - DEPRESSION & SCREENING - PREGNANCY/ANTENATAL/POSTNATAL PERIOD] (1090)

42 exp Animals/ not (exp Animals/ and Humans/) (16785381)

43 41 not 42 [ANIMAL-ONLY REMOVED] (711)

44 (comment or editorial or news or newspaper article).pt. (1788984)

45 (letter not (letter and randomized controlled trial)).pt. (1968033)

46 43 not (44 or 45) [OPINION PIECES REMOVED] (709)

47 46 use medall [MEDLINE RECORDS] (363)

48 exp Depressive Disorder/ (500056)

49 depress*.tw,kw. (1256850)

50 dysthym*.tw,kw. (10863)

51 blues.tw,kw. (4502)

52 melanchol*.tw,kw. (11638)

53 MDD.tw,kw. (34353)

54 PND.tw,kw. (11109)

55 PPD.tw,kw. (22001)

56 or/48-55 [GENERAL DEPRESSION] (1448092)

57 screening/ (278857)

58 mass screening/ (149657)

59 screening test/ (67501)

60 (screen* or detect*).tw,kw. (6034447)

61 (identif* or recogni*).ti. (782823)

62 ((early or earlier or earliest) adj5 (identif* or recogni*)).tw,kw. (159175)

63 (case finding? or casefinding?).tw,kw. (12002)

64 or/57-63 [GENERAL SCREENING] (6817028)

65 depression assessment/ (843)

66 56 and 64 (147122)

67 65 or 66 [DEPRESSION & SCREENING/ASSESSMENT] (147782)

68 randomized controlled trial/ or controlled clinical trial/ (1219653)

69 exp "clinical trial (topic)"/ (231597)

70 (randomi#ation? or randomi#ed or randomly or RCT$1 or placebo*).tw,kw. (2166401)

71 ((singl* or doubl* or trebl* or tripl*) adj (mask* or blind* or dumm*)).tw,kw. (395018)

72 trial.ti. (455346)

73 or/68-72 (2890315)

74 67 and 73 [RCTs - DEPRESSION & SCREENING/ASSESSMENT] (15937)

75 exp pregnancy/ (1594897)

76 exp pregnancy disorder/ (536842)

77 exp named groups by pregnancy/ (87686)

78 pregnan*.tw,kw. (1139799)

79 exp maternal care/ (38982)

80 perinatal care/ (17005)

81 prenatal care/ (60823)

82 postnatal care/ (11313)

83 perinatal period/ (31749)

84 prenatal period/ (9071)

85 puerperium/ (60823)

86 (prenatal* or pre-natal* or antenatal* or ante natal* or antepartum or ante partum or perinatal* or peri-natal* or peripartum or peri-partum or postnatal* or post-natal* or postpartum or post partum or puerperal or puerperium).tw,kw. (771998)

87 (maternal* or maternit*).tw,kw. (617240)

88 expectant mother/ (1157)

89 (expectant mother* or "new mother" or "new mothers" or "mother-to-be" or "mothers-to-be").tw,kw. (11763)

90 or/75-89 [PREGNANCY/ANTENATAL/POSTNATAL PERIOD] (2704011)

91 74 and 90 [RCTs - DEPRESSION & SCREENING/ASSESSMENT - PREGNANCY/ANTENATAL/POSTNATAL PERIOD] (1173)

92 exp animal experimentation/ or exp animal model/ or exp animal experiment/ or nonhuman/ or exp vertebrate/ (46719203)

93 exp human/ or exp human experimentation/ or exp human experiment/ (36484992)

94 92 not 93 (10235912)

95 91 not 94 [ANIMAL-ONLY REMOVED] (1087)

96 editorial.pt. (1004022)

97 letter.pt. not (letter.pt. and randomized controlled trial/) (1963354)

98 95 not (96 or 97) [OPINION PIECES REMOVED] (1085)

99 conference abstract.pt. (3109222)

100 98 not 99 [CONFERENCE ABSTRACTS REMOVED] (940)

101 100 use emczd [EMBASE RECORDS] (443)

102 "Depression (Emotion)"/ (127852)

103 exp Major Depression/ (170646)

104 depress*.tw. (1245207)

105 dysthym*.tw. (10769)

106 blues.tw. (4479)

107 melanchol*.tw. (11518)

108 MDD.tw. (34141)

109 PND.tw. (11036)

110 PPD.tw. (21864)

111 or/102-110 [GENERAL DEPRESSION] (1318110)

112 Screening/ (278857)

113 exp Screening Tests/ (24372)

114 exp Health Screening/ (217092)

115 (screen* or detect*).tw. (6019590)

116 (identif* or recogni*).ti. (782823)

117 ((early or earlier or earliest) adj5 (identif* or recogni*)).tw. (159003)

118 (case finding? or casefinding?).tw. (11839)

119 or/112-118 [GENERAL SCREENING] (6848056)

120 111 and 119 [SCREENING FOR DEPRESSION] (138404)

121 Clinical Trials/ (83742)

122 (randomi#ation? or randomi#ed or randomly or RCT$1 or placebo*).tw. (2162657)

123 ((singl* or doubl* or trebl* or tripl*) adj (mask* or blind* or dumm*)).tw. (394794)

124 trial.ti. (455346)

125 or/121-124 (2411603)

126 120 and 125 [RCTs - SCREENING FOR DEPRESSION] (13280)

127 Pregnancy/ (1518196)

128 Adolescent Pregnancy/ (18952)

129 exp Obstetrical Complications/ (1305)

130 pregnan*.tw. (1113035)

131 Perinatal Period/ (31749)

132 exp Prenatal Care/ (162620)

133 Postnatal Period/ (33492)

134 (prenatal* or pre-natal* or antenatal* or ante natal* or antepartum or ante partum or perinatal* or peri-natal* or peripartum or peri-partum or postnatal* or post-natal* or postpartum or post partum or puerperal or puerperium).tw. (763060)

135 (maternal* or maternit*).tw. (607664)

136 Expectant Mothers/ (612)

137 (expectant mother* or "new mother" or "new mothers" or "mother-to-be" or "mothers-to-be").tw. (11755)

138 or/127-137 [PREGNANCY/ANTENATAL/POSTNATAL PERIOD] (2531594)

139 126 and 138 [SCREENING FOR DEPRESSION - PREGNANCY/ANTENATAL/POSTNATAL PERIOD] (996)

140 exp Animals/ not (exp Animals/ and Humans/) (16785381)

141 139 not 140 [ANIMAL-ONLY REMOVED] (664)

142 141 use medall,emczd (513)

143 141 not 142 [PSYCINFO RECORDS] (151)

144 47 or 101 or 143 [ALL DATABASES] (957)

145 remove duplicates from 144 (571) [TOTAL UNIQUE RECORDS]

146 145 use medall [MEDLINE UNIQUE RECORDS] (361)

147 145 use emczd [EMBASE UNIQUE RECORDS] (170)

148 145 not (146 or 147) [PSYCINFO UNIQUE RECORDS] (40)

***************************
