# Supplementary material for: Screening for depression in women during pregnancy or the first year postpartum and in the general adult population: a protocol for two systematic reviews to update a guideline of the Canadian Task Force on Preventive Health Care
Source: Syst Rev. 2019 Jan 19;8:27. doi: 10.1186/s13643-018-0930-3 (PMC6339426; doi:10.1186/s13643-018-0930-3)
Supplement: Supplementary file 6 — Draft screening forms. (DOCX 14 kb) [file 13643_2018_930_MOESM6_ESM.docx]

## **Additional file 6. Draft screening forms for KQ1**

These questions are specific to the pregnancy and postpartum population and will be edited to address the general adult population.

**Title and abstract screening**

1. Does this reference discuss depression screening in women during pregnancy or in the postpartum period (immediately after birth and extending to 12 months)?

**🔿 Yes/unclear***

🔿 No

*Those answered yes/unclear will be passed through to full-text screening.

**Full-text screening**

1. Language of publication

**🔿 English or French**

🔿 Other _____________________

1. Is this article a randomized controlled trial/cluster randomized controlled trial?

**🔿 Yes**

🔿 **Relevant SR**

🔿 Observational design (e.g., narrative review, cohort, case-control, cross-sectional, case series, case report)

🔿 Abstract

1. Does the population include pregnant and postpartum (up to 12 months postpartum) women?

**🔿 Yes**

🔿 No

**🔿 Mixed population (pregnant/postpartum and general population)**

**🔿 Unclear (contact authors)**

1. [If answer to question 3 is mixed population] If this article includes a mixed population, do they provide pregnancy/postpartum specific outcomes data?

**🔿 Yes**

🔿 No

1. Does this study determine patient eligibility and randomize patients prior to administering the screening test?

**🔿 Yes**

🔿 No

**🔿 Unclear (contact authors)**

1. Does the study provide similar depression management and treatment resources to patients who were identified as depressed via screening in the screening arm of the trial and patients in either the screening or non-screening arms of the trial who were identified as depressed via other methods (e.g., unaided clinician diagnosis, patient report)?

**🔿 Yes**

🔿 No

**🔿 Unclear (contact authors)**

Typically, these questions are nested. If an answer allows us to proceed in the inclusion criteria, the next question will appear. Those bolded would be those that would pass through to the following question.
