# Supplementary material for: Screening for depression in women during pregnancy or the first year postpartum and in the general adult population: a protocol for two systematic reviews to update a guideline of the Canadian Task Force on Preventive Health Care
Source: Syst Rev. 2019 Jan 19;8:27. doi: 10.1186/s13643-018-0930-3 (PMC6339426; doi:10.1186/s13643-018-0930-3)
Supplement: Supplementary file 7 — Draft items for data extraction. (DOCX 14 kb) [file 13643_2018_930_MOESM7_ESM.docx]

## **Additional file 7. Draft items for data extraction**

**Publication details:** year of publication, language, publication status

**Characteristics of study**: study design, methods, country, setting, sample size, number of centres [if applicable], duration of follow-up, source of funding

**Characteristics of population**: age, gender/sex*, race/ethnicity, immigrant status, socioeconomic status (e.g., income, level of education), other risk factors for depression, information regarding respondent bias/representativeness of the included population, support status, partum status, geographical location

**Details about the exposure/intervention**: type of screening test performed, screening tool(s)/question(s) used, timing period and frequency of screening

**Details about comparator**: any additional services provided to comparator group

**Outcomes of interest**: definitions, measurement methods, data, adjusted and unadjusted effect estimates

**Risk of bias items**

*for general adult population
