# Supplementary material for: Screening for depression in women during pregnancy or the first year postpartum and in the general adult population: a protocol for two systematic reviews to update a guideline of the Canadian Task Force on Preventive Health Care
Source: Syst Rev. 2019 Jan 19;8:27. doi: 10.1186/s13643-018-0930-3 (PMC6339426; doi:10.1186/s13643-018-0930-3)
Supplement: Supplementary file 8 — Stakeholder review and feedback. (DOCX 38 kb) [file 13643_2018_930_MOESM8_ESM.docx]

**Additional file 8. Stakeholder Feedback: Depression Screening Protocol**

| Questions | Replies | Comments | ERSC Response |
| --- | --- | --- | --- |
| Do the research questions address the clinically important issues? | Yes: 6  No: 0 | 1: I also think that there is a need to consider the validity and acceptability of screening tools within the context of Indigenous populations. There are well documented differences in communication styles, as well as cultural influences in how questions are viewed and interpreted. It is not just a question of the benefits/harms of screening, but also the appropriateness of the screening tool that is at issue.  2: Yes, the research questions address clinically important issues.  3: I don’t know if adherence to medication (for other chronic illnesses) as a clinically important issue was captured i.e. Depression as a prediction of adherence to antiretroviral therapy in HIV positive pregnant women. I don’t know if stigma or cultural interpretations are mentioned or addressed in any of the reviews.  4: The rationale provided supports the objectives or questions for these SRs. The methodology used is consistent with the guidelines for performing these SRs including the inclusion and exclusion criteria and others.  5: No comments. | 1: Thank you, this is a good point to consider. We will be capturing different populations from the trials, however, the appropriateness, acceptability, and validity of screening tools is outside of the working group’s scope due to limited timelines and resources.  3: Thanks, the working group has rated a list of outcomes and attempted to limit to 7 outcomes to satisfy GRADE methodology recommendation. Adherence to other medications may be captured as a risk factor for depression depending on the reporting of studies. With the additional feedback from the patients, labelling/stigma were considered important and have been added as outcomes for both populations. |
| Are there any important sources of evidence that we did not include that should be considered in our review?  If yes, please provide the source. | Yes: 0  No: 6 | 1: None that I can think of at present, though I am not sure that limiting the sources of grey literature to such a few organizations will capture the breadth of evidence that may be available.  2: The list of data sources was very comprehensive. I can’t think of any that additional ones to include.  3: In reviewing Association websites and guidelines in the Grey Literature - I wonder if it would also be relevant to include other health professional sites such as Midwifery, Nursing, Occupational Health and also Pharmacy  4: The most important study populations have been considered.  5: No comments. | 1: We are also using the CADTH Grey Matters checklist, which includes several additional websites. We have just supplemented this checklist with additional websites.  3: Thanks, we have added the Canadian Nurses Association, the American Nurses Association, and the Canadian Association of Midwives. For occupational health and pharmacy associations, we could not locate on their sites any relevant material for the review (e.g., publications, reports). |
| Do you have any major concerns about the protocol that we should address? | Yes: 2  No: 4 | 1: I am a bit concerned that the protocol may not lead to evidence that reflects Indigenous peoples’ perspectives on screening for depression because of its heavy emphasis on RCTs and what is traditionally considered as ‘evidence’ in western medical research, with its focus on biomedical approaches. There have been very few RCTs done with Indigenous populations, and so focusing so heavily these as the ‘gold standard’ of evidence is going to lead to evidence that is not particularly relevant for Indigenous peoples. This is especially a concern because rates of suicide are particularly high among First Nations and Inuit people compared the general Canadian population.  2: (i) Recommend revisiting treatment aspects of the intervention vs. comparator groups as the information within Tables 1 and 2 (i.e., study inclusion and exclusion criterion) do not reflect that within Figures 1 and 2.  (ii) Regarding the subpopulation analyses (i.e., those at an elevated risk of depression based on characteristics defined in primary studies) – I suspect this will introduce a significant amount of variability in terms of the at risk population – would it not be better to choose a minimum set of risk factors a priori?  (iii) Given the wide ranging outcomes included, it is unclear how the researchers will deem the screening program as “effective” in order to proceed to assess outcomes on patient values and preferences.  (iv) Outcomes related to patient values and preferences are missing from Tables 1 and 2.  (v) For more minor comments, please see track changes and comments within the attached three documents.  3: Although RCT are considered the gold standard, I wonder of other forms of evidence, such as qualitative studies would provide insight, especially when looking at the harms of an intervention.  4: I have no major concerns at this time.  5: Although not a “major concern”, perhaps examining how culture and SES may or may not influence the results. I appreciate that this is a beginning, however to not examine these factors reduces the applicability a/o utility of the checklist. Inclusion of these factors would make the instrument a stronger one. | 1: Thank you for your comment and raising this issue. There have been working group discussions about which study designs to include, but due to the number of observational studies, we have decided to limit to RCT studies.  2: (i) Tables 1 and 2 will guide the reviewers' selection of relevant studies to include in the review during the project. Whereas Figures 1 and 2 are meant to show broadly the possible clinical pathway and be a conceptual tool to help with the design of the project, but also has key elements of the review (e.g., outcomes).  2: (ii) Thank you, the risks of depression are abundant, and defined differently in different studies. We have delineated groups of patients who should not be screened because they should receive thorough depression assessment as part of standard care. Beyond that, it is not clear a priori what risk factors might be incorporated into a screening program, and we do not know that we will find any examples of this. Therefore, we decided that if researchers to classify an eligible population as high risk for screening, that we will review the evidence as such.  2: (iii & iv) This systematic review is being conducted to inform a guideline on screening for depression. This protocol describes the methods for the first systematic review, which will address KQ1 and KQ1a (effectiveness of screening). We are signaling our intention to conduct a separate systematic review on KQ2 and KQ2a on patient values and preferences should the working group decide it is needed to inform the guideline. This will be decided after reviewing the evidence from the outcomes. If the Task Force working group believes that systematic review information on patient values and preferences would potentially change recommendations, beyond what is known from the KT Team’s focus groups, then we will move forward to do the review. We will follow GRADE methods and weigh all a priori identified critical outcomes for both benefit and harm.  If we do pursue a systematic review on KQ2 and KQ2a (patient values and preferences), a separate protocol will be developed (including the relevant PICO criteria).  2: (v) Track changes addressed below  3: We agree that observational and qualitative studies would contribute to harms outcomes, but due to limited timelines, it has been decided that the highest quality of evidence, in RCTs, would be considered and include any harms outcomes.  4: Thank you.  5: Thanks, we plan to look at socioeconomic status and race/ethnicity for our subgroup analyses for both systematic reviews. |

Specific Comments in the Protocol

| Line | Excerpt | Stakeholder Comment | ERSC Response |
| --- | --- | --- | --- |
| 79 & 207 | Depression is the most common cause of disability worldwide, with over 300 million people currently living with depression. | According to GBD 2016 - mental disorders overall (as opposed to depression alone) are ranked #1 in terms of years of life lived with a disability. | Thank you, we have edited this section. We used the prevalence of depression from the 2018 WHO fact sheet, rather than the disability adjusted life years. |
| 80 | (same as above) | Reference? | References are not recommended in Abstracts and the reference is cited in the main text. |
| 87 | … the Cochrane Library | For original studies presumably? | Yes. The search with the Cochrane Library allows for a search of the primary studies within the SRs. |
| 99 | … false positive result… | Screen? | This would be a positive result based on screening, with a negative result when further diagnostic assessment if performed. |
| 101 | These two systematic reviews will offer informative evaluations of depression screening. The findings will be used by the CTFPHC to help develop guideline recommendations on depression screening in pregnant and postpartum women and the general adults population in Canada. | Not clear how the results of these systematic reviews will achieve this. | The findings of the reviews will be based on the critical and important outcomes selected by the working group and patients. These results will be presented following GRADE methodology and reviewed by the working group to prepare their guideline recommendations. More details on the process is available in the CTFPHC Procedure Manual (<https://canadiantaskforce.ca/methods/>) |
| 120 | This protocol outlines the methodological process for synthesizing systematic reviews (SR)… | Up until this point, I presumed this protocol was a systematic review of original studies however, this statement suggests that a review of systematic reviews will be undertaken. | Changed the word to “performing” instead of synthesizing. |
| 135 | The DSM-5 allows for a specifier for depressive episodes that have their onset in pregnancy or within four weeks postpartum, collectively termed major depressive episodes, with peripartum onset. | There is no mention of this in ‘Additional file 1’ unless I’m overlooking it! | Additional File 1 is titled “Additional file 1 DSM-5 and ICD-10 definition of MDE.docx” |
| 172 | More recently, in a study from the US where women were interviewed, and diagnosis made using the DSM-IV criteria, the 12-month period prevalence of major depressive disorder (MDD) was 8.4% among pregnant women, 9.3% among postpartum women, and 8.1% among non-pregnant women [16] | Consider highlighting differences b/w the AHRQ SR and recent study in the US that could explain why the prevalence of depression varied so greatly. | The original number presented was the point prevalence. We have modified this number to be the period prevalence (from conception to birth and from birth to 3 months postpartum), as this aligns more closely to what is reported in the second reference. We have added extra text, as those considered postpartum in the Vesga-Lopez study could have been pregnant during the reporting period, as they report 12-month period prevalence. Whereas the AHRQ study considered the post-partum period from birth to 3 months after delivery. The resulting prevalence rates are much closer. |
| 199 | Typically, it varies by each provincially developed program | What about the territories? | Nunavut has been added to the list in the main document, with a description and link to their program in Additional file 2. |
| 213 | The 2012 Canadian Community Health Survey-Mental Health used the diagnostic interview technique among 25,113 individuals and reported annual prevalence for MDD of 3.9% (95% CI 3.5-4.2%) and lifetime prevalence of 9.9% (95% CI 9.3-10.5%) [31] | These estimates are not from the 2012 CCHS Mental Health Survey and the reference is incorrect. Recommend citing and using the estimates reported within the following paper:  https://www150.statcan.gc.ca/n1/pub/82-624-x/2013001/article/11855-eng.htm | Apologies, this was referenced wrong. It should reference the 2015 Patten paper. This Statcan link references the same numbers as does Patten 2015. |
| 215 | It also reported an annual and lifetime prevalence of MDE among Canadians at 4.7% and 11.3%, respectively [31]. | These estimates are not from the referenced study: S. B. Patten et al., “Descriptive epidemiology of major depression in Canada,” Can J Psychiatry, vol. 51, no. 2, pp. 84–90, Feb. 2006 | Thank you for catching this. The numbers are taken from the 2015 Patten paper. The reference has been updated. |
| 231 | Effective interventions to reduce the effects of depression exist, once one is identified. The Mental Health Commission of Canada reports that almost half of those who feel they have suffered from depression or anxiety have not seen a doctor about this problem [9]. In addition, among those who have been diagnosed accurately, many do not receive minimally adequate treatment [11], [12]. | This lead in doesn’t relate to the remainder of the paragraph i.e., the wide ranging impacts of depression in the general population. | I can appreciate that this isn’t clear. I have reworded the first sentence to relate more to the remainder of the paragraph.  “Although effective interventions to reduce the effects of depression exist, one needs to be identified to benefit from these interventions.” |
| 238 | Depression has been linked to higher chronic disease rates. Once a person is ill, depression can be associated with greater morbidity and mortality. For example, depression has been associated with cardiovascular disease (CVD), and those with CVD who are depressed are at greater risk for recurrent cardiovascular events and mortality [37]. | May want to touch on the bidirectional relationship between depression and other (physical) chronic conditions. | Thank you for this comment. We have edited this section as follows:  “A review by Evans et al. [19] conclude that there may be a bidirectional link between depression and disease, as depression might be an etiologic factor for incident disease (e.g., cardiac disease, stroke) and also might affect the course of medical diseases (e.g., cardiac, diabetes).” |
| 244 | … the total economic burden of mental illness (including health service utilization, long- and short-term work loss, and health-related quality of life) was said to be $51 billion dollars [40]. | Possible to report on the direct and indirect costs associated with depression alone using more recent data? | We have added some newer data that looks at per-capita healthcare cost and excess cost of MDD compared to those without MDD or psychological distress. Although the data is for Ontario, it does provide some evidence that there are extra healthcare costs among those with MDD. |
| 264 | What are the benefits and harms of screening for depression during pregnancy and up to one-year postpartum in primary care or other non-mental health clinic settings | May want to include an example of a non-mental health clinic setting. | Details on what is considered a non-mental health setting can be found in both PICOs tables under the Setting row.  For the pregnant and postpartum review, we specified non-mental health clinic settings as including specialty clinics such as obstetrical, maternal-fetal medicine, and pediatric clinics. For the general adults, we specified as rheumatology, obstetrics and gynecology. |
| 271 | Based on the evidence from both key questions, if screening is found to be effective, and the CTFPHC is interested in a key question on patient values and preferences. | - How will you decide that screening is effective or not?  - You mention “a” key question here and yet there are a few outlined below.  - I don’t understand the numbering system used for the key questions on this page. | This has been addressed in the above section in response 2 (iii & iv). Additional text has been added to the protocol to describe this process. |
| 273 | How do patients value outcomes that may occur from screening for depression during pregnancy and up to one-year postpartum and how do these values influence decisions about being screened? | Unclear how this will be assessed/measured. | Addressed above in response 2 (iii & iv). |
| 300 | A Depression Working Group of CTFPHC members… | How were these members selected? Consider including a list of members and their affiliations in the additional file. | Please see the CTFPHC Procedure Manual (<https://canadiantaskforce.ca/methods/>) for information on selection of working group members and clinical experts. The authors of the protocol are available in the authorship list. |
| 303 | … input from external clinical and content experts | How were the experts selected? Consider including a list of members and their affiliations in the additional file. | Please see the CTFPHC Procedure Manual (<https://canadiantaskforce.ca/methods/>) for information on selection of working group members and clinical experts. The authors of the protocol are available in the authorship list. |
| 314 | All patient-important outcomes will be rated as critical… | By whom? | Edited this sentence for clarification. Patients will be rating the outcomes according to the GRADE methodology. |
| 321 | We will also use the conduct reported in a Measurement Tool to Assess the Methodological Quality of Systematic Reviews | Again, this suggests you’re doing a SR of reviews? | We are using this as a best practice tool to ensure that we meet the quality of conduct criteria as defined in AMSTAR 2. |
| 325 | … Table 1 and Table 2 | Both tables are missing the language restrictions. | This line has been added to both tables.  “English and French” |
| 328 | - If >80% of women have a recent history of depression…. | Why 80%? | This should be 20%, as to ensure that a large representation of the included population have had no recent history of depression. This edit has been made in both PICOs tables. |
| 328 | *characteristics as defined in primary studies | I suspect this will introduce a significant amount of variability in terms of the included studies. | It was decided that this will be kept open to review what is in the available literature. Attempts to categorize risks in relation to specific risk factors will have the added complication of blurring the distinction between screening and clinical management. |
| 328 & 330 | Interventions that, in addition to screening, include depression care referral or treatment options that are not available to patients identified as depressed in the no screening trial arm | I’m confused by this as in figure 1 it appears that the intervention group should receive treatment however, the comparator group does not. | Figure 1 shows the clinical pathway of depression screening, diagnosis and treatment not the pathway of trial design. |
| 328 & 330 | Outcomes | Outcomes on patient values and preferences missing. | Addressed above in response 2 (iii & iv). |
| 330 | - If >80% of women have a recent history of depression | Note, same exclusion criterion as in pregnant and post-partum review. Should it be modified to reflect the general population i.e., women and men? Also, why > 80%? | This should be 20%, as to ensure that a large representation of the included population have had no recent history of depression. This edit has been made in both PICOs tables. |
| 338 | There will be no date or language restriction in the search. | There is for the adult population review i.e., Published from May 2012 until the date the search is run. | Additional words have been added to clarify the search dates for each review. |
| 357 | For the pregnant and postpartum review, we will also search the following websites: | I presume that all of the aforementioned sources will be searched for both SR and that the ones mentioned here are in addition to those. | Correct. I have added some wording to make this clearer. |
| Figure 1 |  | What does the “1” in the circle supposed to indicate in the flow chart below? | “1” in Figure 1 includes key question 1 and the sub-key question 1a and is described in the legend. |
